# Development of a Bio-Inspired Aerogel with Robust Sustainability and Thermal Insulation Performance

**DOI:** 10.3390/ma18122808

**Published:** 2025-06-15

**Authors:** Zhao Zhang, Ying Wang, Maiyong Zhu, Songjun Li

**Affiliations:** School of Materials Science & Engineering, Jiangsu University, Zhenjiang 212013, China

**Keywords:** aerogels, tendon, sustainability, thermal insulation performance

## Abstract

The aim of this study was to develop a practical aerogel with robust sustainability and thermal insulation performance. Inspired by the muscular movement of creatures, this target was achieved by developing a tendon-like composite aerogel. The composite aerogel provided comprehensive reinforcement due to the comparable composition and compatibility, which made the robust sustainability and thermal insulation performance feasible. The results showed that the prepared aerogel exhibited the desired sustainability and thermal insulation performance, manifesting better abrasion resistance (increased by up to 12.3 times) and lower thermal conductivity (decreased by 25.9%) in contrast to the counterpart in the absence of the tendon. The adoption of the tendon-inspired design also ameliorated the physicochemical properties, including the higher specific surface area (2673.64 m^2^·g^−1^), porosity (94.12%) and hydrophobic properties (138.1°). The robust sustainability and thermal insulation performance of the prepared aerogel, coupled with the better physicochemical properties, indicate the prospect of developing sophisticated aerogels for applications, particularly outdoor applications involving a rapid change of heat.

## 1. Introduction

Studies on aerogels and their thermal insulation performance are undergoing exponential expansion by virtue of their versatile usability [1,2]. Aerogels originate from polymeric gel networks but have the liquid media replaced with gas. For this reason, aerogels may offer significant advantages compared to conventional polymeric gels, typically manifesting high porosity [3,4]. The high porosity makes the low weight density and the high surface area feasible, which grants the excellent thermal/acoustic insulation performance. One prominent ongoing application would be silica aerogels, which arguably provide terrific thermal insulation performance due to the robust Si-O-Si links and firm polymeric networks [5]. The robust Si-O-Si links and firm polymeric networks may tolerate rapidly changing temperatures. British “Puma” fighters, the Russian “Mir” space station, the American “Mars Rover” and other aerospace vehicles have all used silica aerogels as thermal insulation materials to resist the rapidly changing temperatures inside/outside the aircraft [6]. Owing to these advantages, silica aerogels are being increasingly adopted in applications, including novel aerospace vehicles, metallurgy, the chemical industry, medicine and health, etc. [7,8].

As far as the preparation methods are concerned, Kistler et al. [9] were the earliest users of sodium silicate as the source of silicon under supercritical drying conditions, in which aerogels were defined as materials obtained from the supercritical drying of wet gels. Nonetheless, taking the complexity, danger and high cost of the preparation processes into account, the excellent thermal insulation performance of silica aerogels has not drawn enough attention from scientific communities. It was not until the 1980s that silica aerogels became easily available and widely adopted materials due to the use of the sol–gel method reported by Bernard et al. [10], in which the prepared silica aerogels were successfully tested as a rocket fuel insulation reservoir. After decades of development, and particularly the development over the last two decades [11,12], the adoption of emerging drying technology and methods have made silica aerogels civilian materials.

The development of aerogels often requires that the prepared aerogels possess robust sustainability during intensive applications. From the view of point of practical usability, aerogels are also required to not be easily worn and damaged by these applications. Unfortunately, the currently available aerogels cannot meet the desired standard and can be easily worn and damaged due to the (firm but) rigid polymeric networks. For this reason, many attempts have been made over the years and impressive advances have been achieved [13,14]. One remarkable achievement of these experiences is the development of composite aerogels [15], which may help ameliorate the porosity and related physiochemical properties of the resulting silica aerogels. Nonetheless, the development of practical aerogels remains a challenge in terms of streamlining the sustainability and thermal insulation performance [16]. The reason for this can be ascribed to the unpredictability of the resulting aerogels, that is, the increasing sustainability does not necessarily lead to better thermal insulation performance due to the changing porosity distribution in the resulting aerogels. It is known that the smaller pores, the larger the specific surface area and the higher the porosity, which provides better thermal insulation performance but not necessarily greater sustainability [17]. The smaller pores, the larger specific surface area and the higher porosity favor the thermal insulation performance due to the more obstacles and resistance against heat transmission and air convection, which leads to the less heat transfer. The larger specific surface area allows more surfaces inside the aerogels to reflect/scatter heat and thus better isolates the heat transmission. The higher porosity indicates a larger number of pores in the aerogels, increasing the pathway for heat conduction and accordingly decreasing the transfer of air convection. As such, many peers and ongoing research studies reveal it to be challenging or even impossible to directly correlate the sustainability with the thermal insulation performance of the resulting aerogels [18,19,20]. New methods and technologies are required for developing practical silica aerogels.

Nature has been humanity’s tutor. For centuries, humanity has been acquiring knowledge from nature. A body of knowledge is now available. One part of this body concerns the tendons [21,22], which share a promising prospect with the struggling field of aerogels. Tendons, composed of collagen fibers and proteoglycans that are involved in diverse interactions, exist with high mechanical strength and robust sustainability. The high strength and robust sustainability of tendons allow creatures to make a variety of movements and even to lift up the creatures themselves. Owing to the chemically comparable composition, the moving muscles have good compatibility with the tendons, which allow muscular movement to move forward or to be stopped immediately without damage. In this way, tendons and their collagen fibers provide backups for muscular movement. Although the known natural phenomenon is not related to aerogels and their applications, the creation of tendon-like functions in aerogels provides a promising solution capable of causing robust sustainability and better physiochemical properties.

**Scheme 1:**
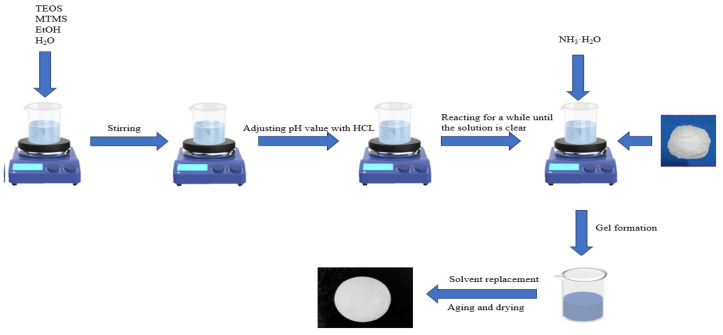
Schematic presentation of the preparation of the proposed aerogel.

Inspired by the exquisite principles of nature, here, we report the development of a tendon-like composite aerogel capable of robust sustainability and thermal insulation performance. Taking the compatibility of the silica matrix in the prepared aerogel into account, glass fiber, due to the comparable composition, was adopted to create the tendon-like function. To this end, this aerogel was developed in accordance with the bio-inspired design (Scheme 1; the details of the preparation process will be presented below). After curing and aging, the proposed aerogel was thus prepared, which allowed the tendon to provide comprehensive reinforcement to the aerogel. The successful preparation of this aerogel indicates the prospect of developing sophisticated aerogels for applications, particularly outdoor applications involving in a rapid change of heat.

## 2. Experimental Section

### 2.1. Materials

The chemicals used were of analytical grade and used as received. The ethyl orthosilicate (TEOS, 98%), methyltrimethoxysilane (MTMS, 97%) and fiber powder (glass fiber, 6.5 μm) were purchased from Shanghai Maclin Biochemical Co. (Shanghai, China). The hydrochloric acid (HCl) (1.0 M), anhydrous alcohol and ammonia (~25 wt-%) were commercially available from Sinopharm Group (Hong Kong, China).

### 2.2. Preparation of Silica Aerogels

The preparation process for the proposed aerogel is schematically presented in Scheme 1. Using the sol–gel method, ethyl orthosilicate (1.4 mL) and methyltrimethoxysilane (2.7 mL) were first dissolved into a mixture of anhydrous alcohol (5.8 mL) and deionized water (9.0 mL). A pH meter was subsequently used to detect and adjust the pH value of the mixture to 2–3 with HCl [23], which allowed the preliminary sol to form. After the mixture system became transparent, the fiber powder (0.3 g) was added. Following this, the pH meter was used again to detect and adjust the pH value of the system to 8.9 with ammonia [24], which allowed the gel to form (i.e., the aerogel precursor). The prepared aerogel precursor was sealed for aging (4 h), followed by solvent exchange in anhydrous ethanol (24 h), in which the ethanol was replaced every 8 h. The resulting aerogel was sealed again and then dried at 60 °C (named “SA-F”, in which the use of the glass fiber is marked with “F”). For a contrastive purpose, the control aerogel was prepared under comparable conditions but without using the glass fiber (named “SA-N”, in which “N” indicates the lack of the fiber).

### 2.3. Analysis and Characterization

The analysis and characterization of the prepared aerogels were carried out with modern tools. The Fourier transform infrared (FTIR) spectra were scanned with a Nicolet iS10 Spectrophotometer (Thermo Fisher Molecular Spectroscopy, Inc., Waltham, MA, USA). The scanning electron microscope (SEM) and transmission electron microscope (TEM) images were taken with a ZEISS Gemini SEM 360 (Carl Zeiss AG, Baden-Württemberg Oberkochen, Germany) and a JEM-2100 apparatus (lectronics Co., Ltd., Tokyo, Japan), respectively. The X-ray diffraction (XRD) patterns were detected using a SmartLab XRD instrument fitted with a rotating anode X-ray source (with the following range: 10–90°) (Rigaku Corporation, Tokyo, Japan). The energy dispersive X-ray (EDX) spectra and elemental mapping were recorded using an H-7650 energy-dispersive spectrometer (Hitachi Corporation, Tokyo, Japan).

### 2.4. Surface Areas and Porous Parameters

The BET specific surface areas and porous parameters were acquired using an ASAP 2460 Surface Area and Porosity Analyzer (Thermo Fisher Molecular Spectroscopy, Inc., Waltham, MA, USA). The measurements were conducted at 77 K. The aerogel samples (0.1 g) were degassed at 100 °C for 24 h and then the data concerning the surface area and porous parameters were determined using an online data system, in which the Barrett–Joyner–Halenda method was used to calculate the porous distribution of the isotherm desorption branches [25].

### 2.5. Contact Angles

The contact angles were detected using a JCY-2 apparatus (ShangHai FangRui Instrument Co., Ltd., Shanghai, China), in which 5 μL of water droplets was dropped onto the surfaces of the prepared aerogels (0.1 g). After lying still until reaching equilibrium, the contact angles of the prepared aerogels were detected using an attached imaging system [26]. The contact angles were then calculated from the average of six runs.

### 2.6. Abrasion Resistance

The abrasion resistance of the prepared aerogels was investigated using sandpaper friction testing [27]. The surface of the prepared aerogel was rubbed for up to 10 times with 800-grit sandpaper. The rubbed surface of the aerogel sample was rinsed with anhydrous ethanol and then dried using a hairdryer (40 °C, 10 min). The contact angles of the prepared aerogels were determined.

### 2.7. Thermal Conductivity

The thermal conductivity of the prepared aerogel samples was measured at room temperature using a Hot Disk TPS 2500S Thermal Constants Analyzer (Hot Disk AB, Gothenburg, Sweden). The measurements were conducted using the transient plane source method, with four parallel tests performed at each temperature point in an air atmosphere, and the test data were recorded in real time through an online data acquisition system [28].

### 2.8. Thermogravimetric Analysis

The thermogravimetric (TG) analysis and the differential TG (DTG) analysis were performed using a STA449F3 Simultaneous Thermogravimetric Analyzer (NETZSCH-Gerätebau GmbH, Wardkraiburg, Germany). The aerogel samples were heated from room temperature up to 800 °C at a rate of 10 °C min^−1^. The changing mass of the aerogel samples was recorded with an attached data system.

## 3. Results and Discussion

### 3.1. Composite and Constitution

**Figure 1:**
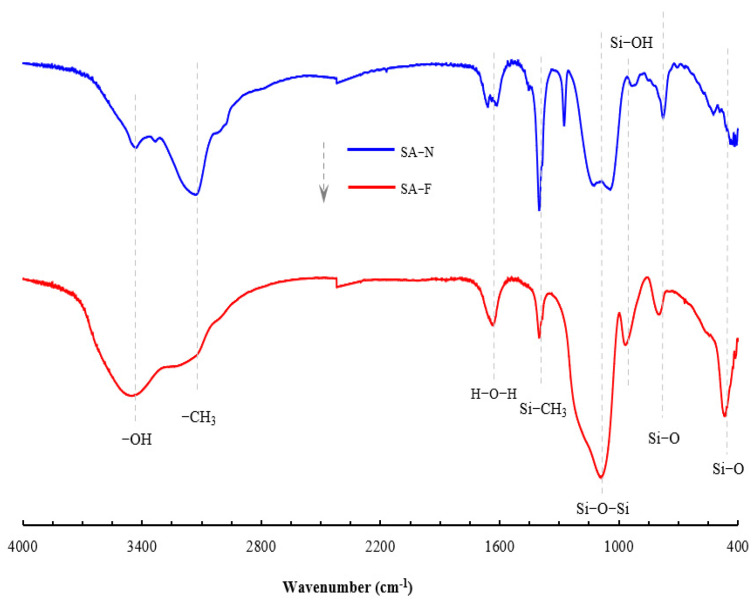
FTIR spectra of the prepared aerogels.

**Figure 2:**
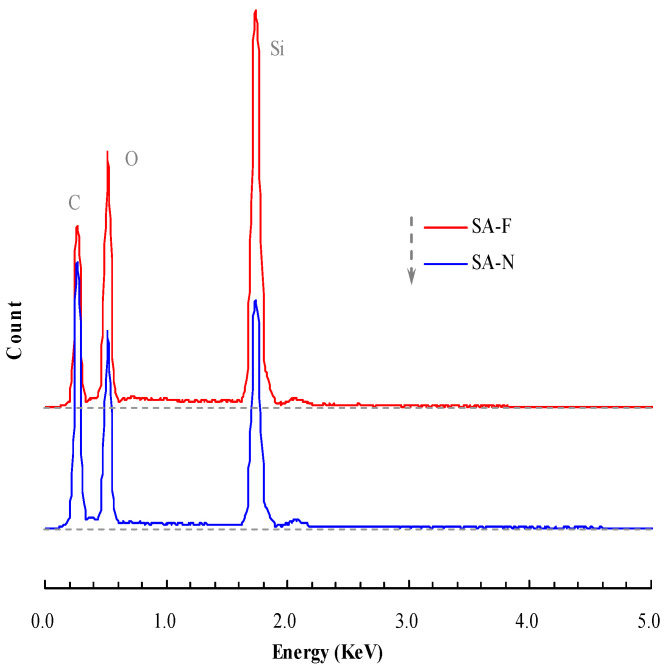
EDX spectra of the prepared aerogels.

**Figure 3:**
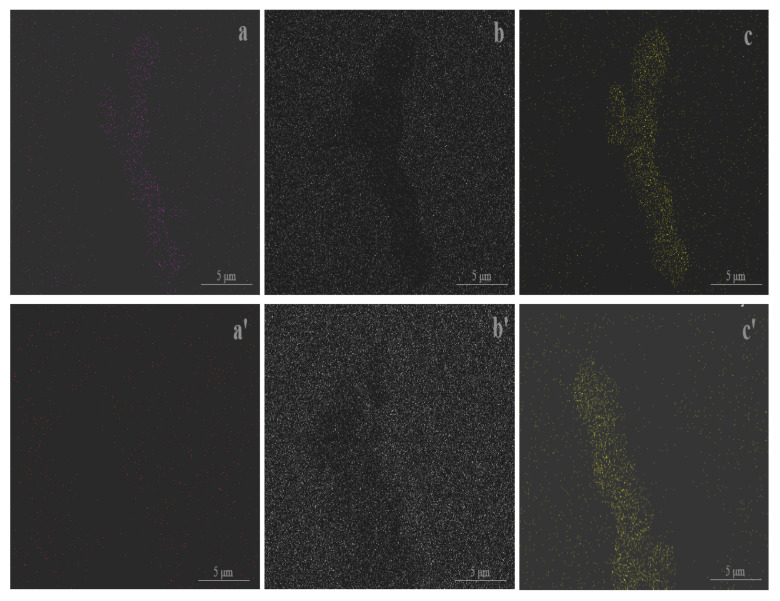
Elemental mapping of the prepared aerogels (from **a**,**a’**–**c**,**c’**: Si, O, C in SA-F (and SA-N)).

**Figure 4:**
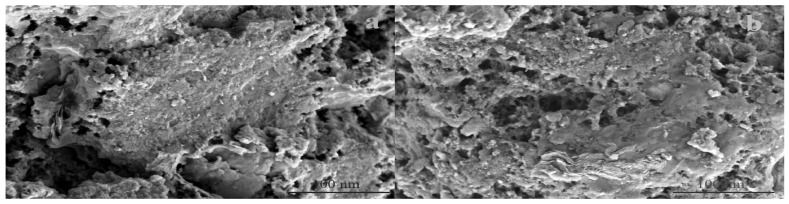
SEM images of the porous constitution of the prepared aerogels (**a**: SA-N; **b**: SA-F).

As explained, the proposed aerogel was developed in accordance with the inspired design. Figure 1 presents the FTIR spectra of the proposed aerogel. For a comparative purpose, the control aerogel is also included in the figure. A series of peaks, including 3450 cm^−1^, 2962 cm^−1^, 1638 cm^−1^, 1095 cm^−1^, 955 cm^−1^, 798 cm^−1^ and 466 cm^−1^, appeared in the spectra. According to the FTIR theory [29], the peak at 3450 cm^−1^ may be assigned to the asymmetric stretching vibration of O-H in the structural water. The peak near 2962 cm^−1^ can be ascribed to the stretching vibration of C-H in the methyl group. The peak near 1638 cm^−1^ may be associated with the bending vibration of water’s H-O-H. The peak at 1095 cm^−1^ may be due to the asymmetric stretching vibration of the Si-O-Si bond, and the peak at 955 cm^−1^ may belong to the bending vibration of Si-OH. The peaks at 798 cm^−1^ and 466 cm^−1^ may be attributed to the symmetric stretching vibrations of the Si-O bond. In contrast, the control aerogel did not show a substantive discrepancy in the spectra, but it did exhibit a difference in the relative peak height. This indicates the presence of the tendon-like fiber in the proposed aerogel, which led to a change in the spectra (further discussion of the fiber and the compatibility will be presented below). The EDX spectra (Figure 2) and elemental mapping (Figure 3) show that each detected characteristic X-ray photon generates a bright spot at the corresponding position. The intensity of the characteristic X-rays is represented by the brightness, with brighter areas indicating higher element concentrations. The measured element content represents the average value of the scanning area, which agreed with the FTIR spectra and revealed the corresponding composition of the elements. Figure 4 displays the porous constitution of the prepared aerogels. The proposed aerogel showed a relatively rougher surface compared to the control aerogel. In conjunction with the preparation (cf. Section 2.2), this outcome indicates that this aerogel was prepared in the proposed form, which accordingly facilitated further studies.

### 3.2. Contact Angles and Abrasion Resistance

**Figure 5:**
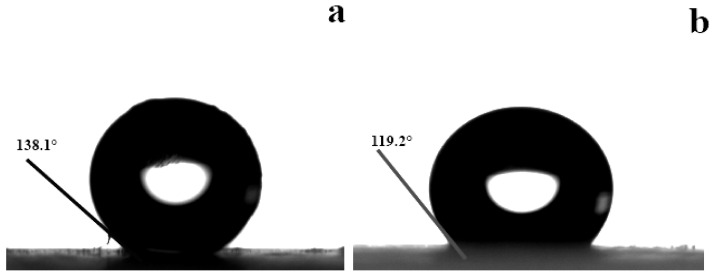
Contact angles of the prepared aerogels (**a**: SA-F; **b**: SA-N).

**Figure 6:**
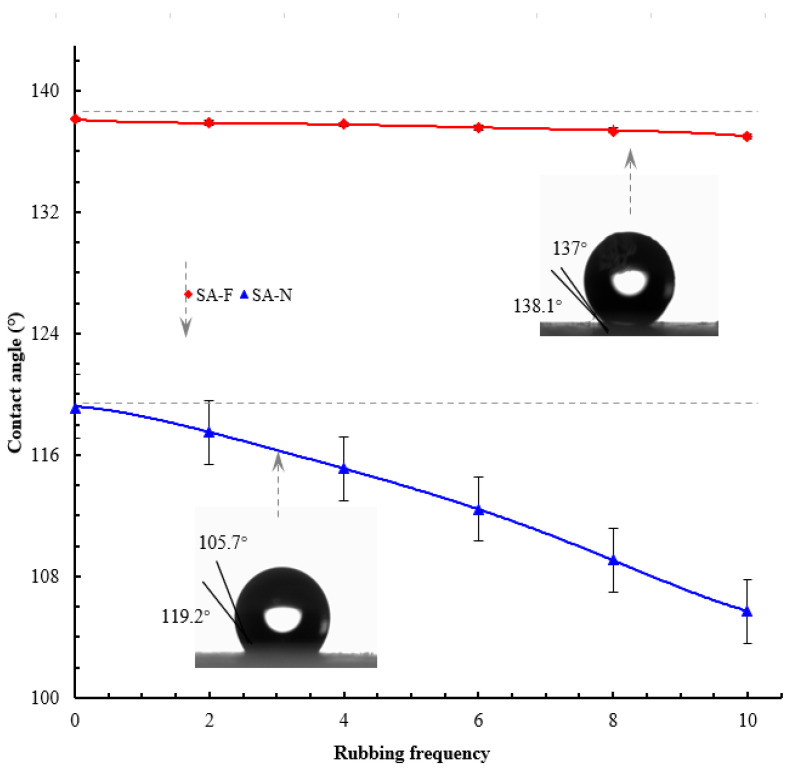
The abrasion resistance with sandpaper rubbing.

Figure 5 presents the contact angles of the prepared aerogels. The proposed aerogel provided a contact angle as high as 138.1°. In contrast, the control aerogel manifested a contact angle of only 119.2°. The adoption of the bio-inspired design for the proposed aerogel played a role in increasing the hydrophobic properties. The reasons for this can be multiple, but the main one, as is commonly known [30] and described in Section 3.1, may be a consequence of the rougher surface (Figure 4), which largely prevents water droplets from hitting the elaborate textures and thus heavily isolates the surface. As such, the proposed aerogel showed higher hydrophobic properties. Figure 6 presents the abrasion resistance of the prepared aerogels, in which the prepared aerogels were repeatedly rubbed against a piece of sandpaper. The proposed aerogel sustained reliable hydrophobic properties after many rounds of rubbing. The repeated rubbing did not lead to a substantive decrease in the hydrophobic properties. Under comparable conditions, the control aerogel resulted in a substantive decrease in the hydrophobic properties. As described in Wenzel’s model [31], a rough surface would lead to the actual contact area being greater than the apparent geometric area, which would increase the surface’s hydrophobicity. However, wear can damage the initial hydrophobic surface and even the exposed hydrophilic groups on the surface, which would conversely decrease the hydrophobic properties. Under the combined effect of the two factors, the hydrophobic properties were reduced, and accordingly, the hydrophobic contact angle decreased. The adoption of the bio-inspired design for the proposed aerogel led to better abrasion resistance (increased by up to 12.3 times; with 10 rounds of rubbing, the contact angle of SA-F decreased from 138.1° to 137° (decreased by 1.1°), while that of SA-N decreased from 119.2° to 105.7° (decreased by 13.5°)). As desired, this could be due to the tendon-reinforced sustainability of the proposed aerogel, which accordingly provided better abrasion resistance.

### 3.3. Porosity and Thermal Insulation Performance

**Figure 7:**
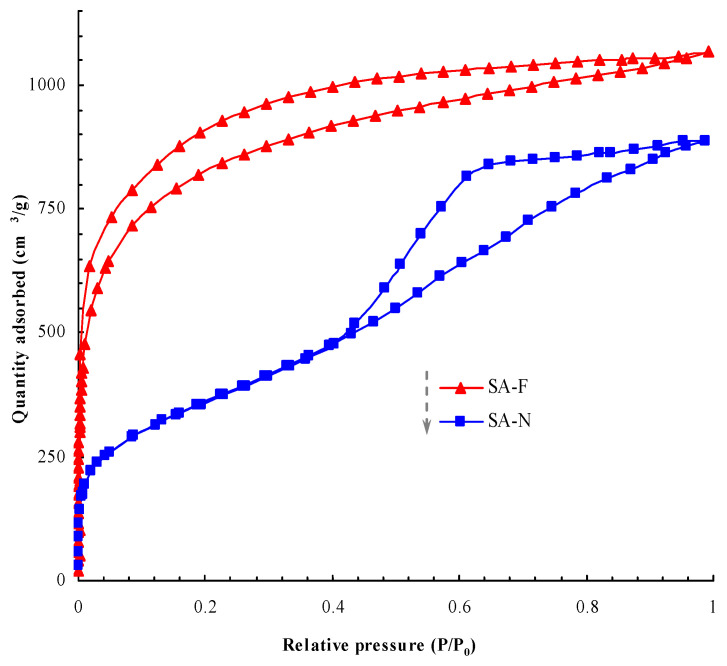
The absorption isotherms of the prepared aerogels.

**Figure 8:**
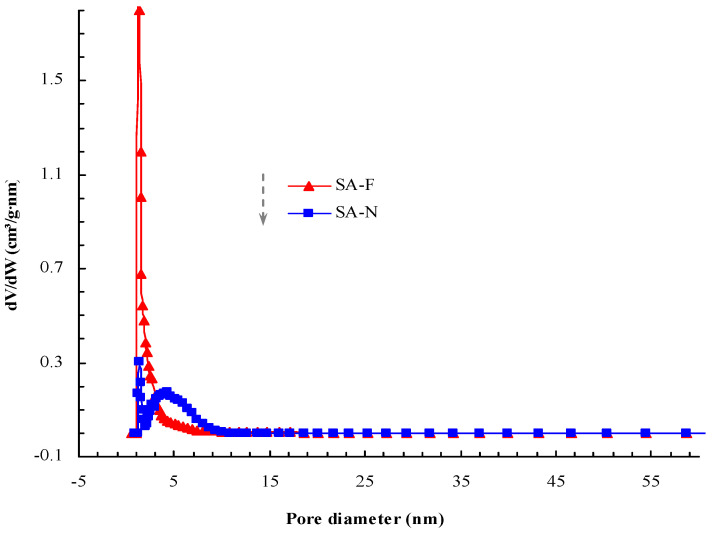
The porous distribution of the prepared aerogels.

**Figure 9:**
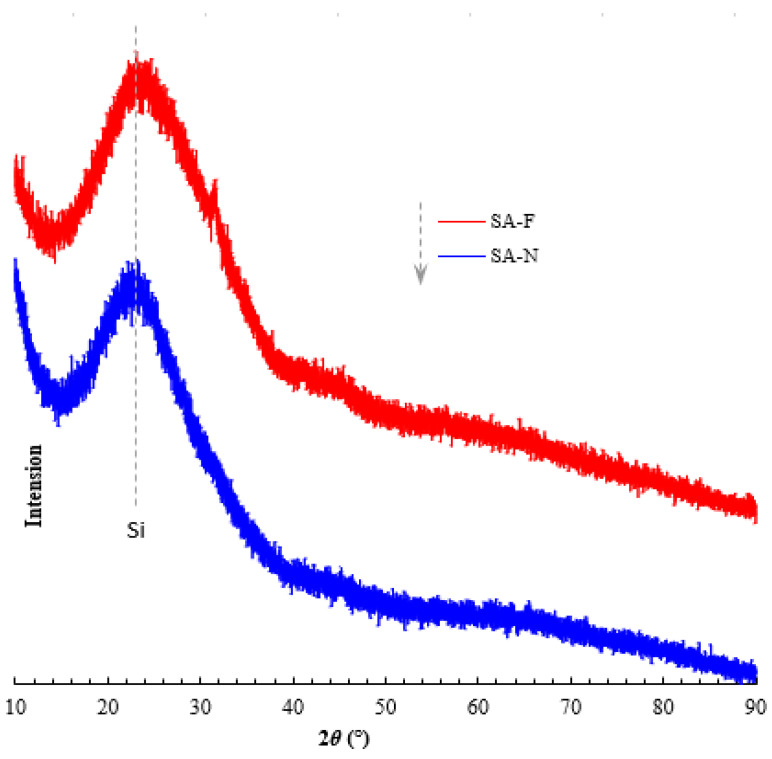
XRD patterns of the prepared aerogels.

**Figure 10:**
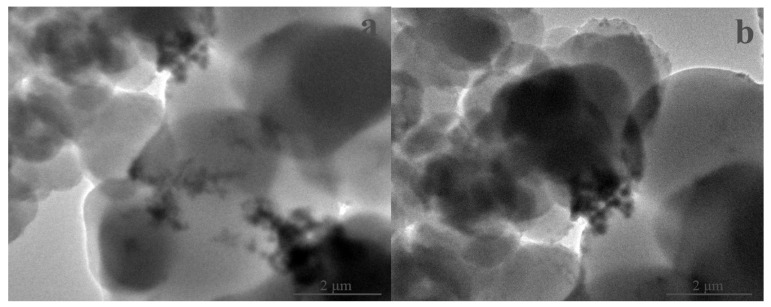
TEM images of the prepared aerogels (**a**: SA-N; **b**: SA-F).

The porosity and related porous parameters are important indexes for evaluating the prepared aerogels. As previously explained (cf. Section 1), the typical porous characteristics would allow the aerogels to exhibit good thermal insulation performance, given the porous transmission pathway that may cause the essential loss of heat and discontinuous transmission. Relatively, the smaller pores, the lager specific surface area and the higher porosity make heat transmission more difficult due to the greater number of obstacles and resistance against heat transmission and air convection, which leads to less heat transfer [18,19,20]. As shown in Figure 7 and Figure 8, the prepared aerogels presented the typical porous properties. Compared with the control aerogel, the proposed aerogel possessed relatively smaller pores, higher surface area, more uniform distribution and higher porosity (Table 1). The higher porosity and porous parameters allowed the proposed aerogel to demonstrate better thermal insulation performance. Fully consistent with the proposed design, the proposed aerogel did show better thermal insulation performance (Table 1). As desired, the adoption of the proposed design for this aerogel played a role in causing better thermal insulation performance. It was noteworthy that no new species was detectable in the proposed aerogel (Figure 9 and Figure 10). Good compatibility was shown between the aerogel and the tendon. As such, the proposed aerogel led to better thermal insulation performance.

### 3.4. Stability and Robustness

**Figure 11:**
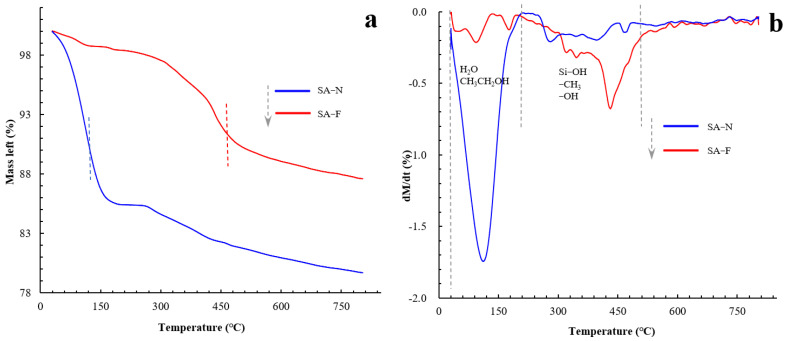
Thermogravimetric analyses of the prepared aerogels (**a**: TG; **b**: DTG).

Using the thermogravimetric analyses, Figure 11 presents the thermal stability of the prepared aerogels. As known, the mass loss observed from room temperature to 150 °C can be ascribed to the evaporation of the adsorbed water and residual solvent. The mass loss between 200 °C and 400 °C may be due to the decomposition of various groups, including the Si-CH_3_, -CH_3_, and -OH moieties. Compared with the control aerogel, the proposed aerogel exhibited relatively slower and significantly reduced mass loss. The proposed aerogel demonstrated relatively better thermal stability compared to the control aerogel in terms of the decomposition temperature. As explained, this may be a consequence of the reinforced sustainability of the proposed aerogel, which exhibited better thermal stability. It is also noted from Figure 11 that the robust sustainability of the proposed aerogel did not substantively change the shape of the TG and DTG curves but led to a shift in the decomposing temperature. In conjunction with the species analyses (cf. Figure 9 and Figure 10; Section 3.3), this outcome indicates again that good compatibility was shown between the aerogel and the tendon. As far as the reason for this was concerned, the glass fiber itself possesses robust polymeric networks [5]. The robust polymeric networks may tolerate rapidly changing temperatures, enabling the proposed aerogel to maintain stability under rapidly changing temperatures. With the composite, the glass fiber may serve as the reinforcing framework, consolidating the aerogels and accordingly preventing the aerogels from collapsing under the rapidly changing temperatures. Furthermore, the fiber can also partially alter the transmission pathway of heat [32], increasing the resistance to heat transfer. These combined effects helped increase the thermal stability of the composite aerogel. As such, the proposed aerogel led to better stability and sturdiness.

## 4. Conclusions

There is much interest in aerogels and their thermal insulation performance by virtue of their versatile usability. The development of aerogels often requires possession of robust sustainability and good thermal insulation performance. Unfortunately, the currently available aerogels cannot meet the desired standard due to the unpredictability of the resulting aerogels, that is, the increasing sustainability does not necessarily lead to better thermal insulation performance due to the changing porous distribution in the resulting aerogels. It is actually difficult to correlate the sustainability with the thermal insulation performance of the resulting aerogels. Inspired by the muscular movement of creatures, a practical aerogel capable of robust sustainability and thermal insulation performance was developed in this study. The adoption of the bio-inspired design provided comprehensive reinforcement to the aerogel, which showed better sustainability and thermal insulation performance, along with better abrasion resistance (increased by up to 12.3 times) and lower thermal conductivity (decreased by 25.9%) compared to the counterpart in the absence of the tendon. Meanwhile, the prepared aerogel also improved the physicochemical properties, including the higher specific surface area, porosity and hydrophobic properties. The specific surface area was as high as 2673.64 m^2^·g^−1^. The water contact angle reached 138.1°, the high porosity was 94.12%, and the thermal conductivity was 41.66 W/(m·K × 10^3^). The prepared aerogel also presented better stability in comparison to the aerogel prepared without the tendon-like design. The successful development of this proposed aerogel may enhance the operational safety and simplify the manufacturing process. This work indicates a promising prospect for developing sophisticated aerogels for applications, particularly outdoor applications involving in a rapid change of heat.

## Figures and Tables

**Table 1:** Porous parameters and thermal conductivity of the prepared aerogels.

	Porosity (%)	BET Surface Area (m^2^·g^−1^)	Pore Volume (cm^3^·g^−1^)	Weight Density (g·cm^−3^)	Thermal Conductivity (W·m^−1^·K^−1^ × 10^−3^)
SA-F	94.12	2673.64	1.62	0.13	41.66
SA-N	86.96	1971.20	1.14	0.12	52.40

## Data Availability

The original contributions presented in this study are included in the article. Further inquiries can be directed to the corresponding author.
